# Effects of Dense Granular Protein 6 (GRA6) Disruption on *Neospora caninum* Virulence

**DOI:** 10.3389/fvets.2020.562730

**Published:** 2020-09-22

**Authors:** Panpan Zhao, Nan Zhang, Jingquan Dong, Jianhua Li, Xiaocen Wang, Xin Li, Xiangrui Li, Ju Yang, Pengtao Gong, Xichen Zhang

**Affiliations:** ^1^Key Laboratory of Zoonosis, College of Veterinary Medicine, Jilin University, Changchun, China; ^2^Jiangsu Key Laboratory of Marine Biological Resources and Environment, Jiangsu Key Laboratory of Marine Pharmaceutical Compound Screening, Co-Innovation Center of Jiangsu Marine Bio-industry Technology, Jiangsu Ocean University, Lianyungang, China; ^3^College of Veterinary Medicine, Nanjing Agricultural University, Nanjing, China

**Keywords:** *Neospora caninum*, dense granules protein 6, intravacuolar network, CRISPR, virulence factor

## Abstract

*Neospora caninum* (*N. caninum*) is a major cause of abortions in cattle. During its invasion of host cells, a parasitophorous vacuole (PV) is formed, accompanied by an intravacuolar network (IVN). The IVN takes part in parasite ingesting of nutrients from hosts. The dense granular proteins of *N. caninum* (NcGRAs) play a key role in forming the PV and the IVN, which may influence virulence during *N. caninum* invasion. The present study aimed to explore the biological function of NcGRA6 in *N. caninum* by disrupting the NcGRA6 gene in the Nc-1 strain. We successfully constructed an NcGRA6-targeting CRISPR plasmid (pNc-SAG1-Cas9:U6-SgGRA6) and amplified the DHFR-TS DNA donor. The NcGRA6 knockout mutation (ΔNcGRA6) was generated by co-electroporation of the pNc-SAG1::CAS9-U6::sgGRA6 plasmid and the DHFR-TS DNA donor into the Nc-1 strain, which was then cultured under pyrimethamine selection pressure. The ΔNcGRA6 mutation was further verified by identification of NcGRA6 gene disruption using PCR, measurement of NcGRA6 gene transcription levels using qPCR, assessment of NcGRA6 protein expression levels using western blotting, and observation of NcGRA6 protein location using immunofluorescence and immunoelectron microscopy. The results of *in vitro* virulence assays, including plaque, invasion, egress, and replication assays, showed that the ΔNcGRA6 strain had smaller plaques, similar invasion and egress ability, and slower intracellular replication ability than the Nc-1 strain. The results of *in vivo* virulence assays showed that the ΔNcGRA6 strain exhibited reduced virulence and improved survival ability in mice compared with the Nc-1 strain. The parasite burden in ΔNcGRA6 strain-infected mouse tissues, including the heart, brain, liver, spleen, lung, and kidney, was significantly reduced compared with that in mice infected with the Nc-1 strain. These data suggest that we successfully constructed a ΔNcGRA6 strain and verify that NcGRA6 is a critical virulence factor. NcGRA6 gene disruption can slow down *N. caninum* proliferation and lower the pathogenicity to hosts. Our findings provide a foundation for future research on other targeted *N. caninum* protein functions and may help in exploring the interaction mechanisms between parasites and hosts.

## Introduction

*Neospora caninum* (*N. caninum*) is an obligate intracellular parasite of the phylum *Apicomplexa* that infects a broad range of hosts and leads to neosporosis worldwide (1–4). Typical clinical symptoms are abortion in dairy cattle and fetal abnormalities and nervous system dysfunction in calves (5, 6). This brings significantly economic losses to the breeding industries (7). However, there are no effective drugs or vaccines available against *N. caninum* (8). After invasion of hosts, tachyzoites of *N. caninum* reside in a specialized membranous organelle known as the parasitophorous vacuole (PV). The PV has the function of resisting host cell acidification and lysosomal zymohydrolysis, which prevents host cells from clearing exogenous parasites. In addition, an intravacuolar network (IVN) enhances parasite ingestion of cytosolic material from the hosts (9). The IVN is made up of highly curved membrane tubules and links parasites and the PV membrane together (10–14).

*Neospora caninum* is closed related to *Toxoplasma gondii* (*T. gondii*). During invasion, parasites continually discharge three types of proteins from different secretory organelles, namely, micronemes (MICs), rhoptries (ROPs), and dense granules (GRAs), either in the process of invasion or afterwards (15). Proteins secreted from MICs could help parasites attach to host cells. Proteins secreted from ROPs could interact with MIC proteins and form a moving junction (MJ) that migrates down the parasite. Proteins secreted from GRAs play important roles in maintaining the PV and IVN structures. Mercier et al. showed that TgGRA6 can stabilize the membrane tubules (16). Bittame et al. reported that the predicted alpha-helical hydrophobic domain of TgGRA6 may be responsible for its connection with the PV and IVN (11). Romano et al. verified that deletion of the TgGRA6 gene led to replacement of the IVN with smaller tubules and vesicles (17). The NcGRA6 gene has been cloned and sequenced, and using a specific polyclonal antibody, its protein were confirmed to have a molecular weight of 37 kDa (18, 19). Although NcGRA6 has similar signal peptides, transmembrane domains, and motifs as *T. gondii*, the homology is as low as 28.76% (20). In *N. caninum*, the GRA6 gene and protein sequences in the low virulence strain Nc-1 and high virulence strain Nc-liv share 100% homology (Figure S1) (21). Thus, research on the function of NcGRA6 would have common significance in different *N. caninum* isolates. NcGRA6 can be used as a diagnostic antigen for detecting serum antibodies in infected animals through ELISA. Meanwhile, it has no cross reactivity with other pathogens, such as *T. gondii*, and *Sarcocystis cruzi*. (22, 23). Thus, it is rather meaningful to study NcGRA6. In our previous study, NcGRA6 was found to exist in soluble and transmembrane forms and to be located in the lumen of the PV and IVN, where *N. caninum* absorbs nutrition and eliminates metabolic waste (20). Previous studies have indicated that the IVN was involved in *T. gondii* virulence (16). Therefore, we inferred that NcGRA6 may be a virulence factor due to its roles in constructing the PV and IVN, which provide safe and metabolically active intracellular compartments to ensure parasites can survive and replicate. Although NcGRA6 is a hot target and several studies have focused on its molecular features, antigenic characteristic, and location distribution, the biological function of NcGRA6 and whether it can influence the pathogenicity of *N. caninum* is still unknown.

In the present study, we constructed an NcGRA6 knockout mutation (ΔNcGRA6) using the CRISPR/Cas9 system and verified the knockout strain at the gene, transcription, and protein expression levels. To explore the pathogenic ability of the knockout mutant strain, virulence assays were conducted both *in vitro* and *in vivo*. Our findings lay a foundation for future research on other *N. caninum* protein functions and provide a new target for drug and vaccine development.

## Materials and Methods

### Ethics Statement

All animal experiments were strictly performed according to the guidelines of the Animal Welfare and Research Ethics Committee of Jilin University (pzpx20190929065). Five-week-old BALB/C female mice were maintained in feeding cages with sterile food, water, and a 12 h light/dark cycle. The mice were used for experiments after an acclimatization period of more than 7 days.

### Cell Culture

MDBK cells (bovine kidney epithelial cells) and HFF cells (human skin fibroblasts) (ATCC, USA) were both maintained in Dulbecco's Modified Eagle's Medium (DMEM, Biological Industries, Ltd., Israel) containing 10% fetal bovine serum (FBS, Biological Industries, Ltd., Israel), 100 U/mL penicillin and 100 μg/mL streptomycin (Biological Industries, Ltd., Israel). Cell cultures were incubated at 37°C under humidified 5% CO_2_ conditions.

### *N. caninum* Culture and Purification

The Nc-1 strain and ΔNcGRA6 strain of *N. caninum* tachyzoites were cultured by continuous passage in MDBK cells. *N. caninum* at the stationary growth phase was collected and centrifuged at 1,000 × g for 10 min after being passed through a 27-gauge needle (Millipore, Billerica, MA, USA). Then, the harvested *N. caninum* tachyzoites were purified with 40% Percoll (2,000 × g, 30 min) and washed with phosphate-buffered saline (PBS, pH 7.4). Finally, the purified *N. caninum* tachyzoites were diluted in cold PBS.

### Generation of the *N. caninum* ΔNcGRA6 Strain

#### Construction of NcGRA6 CRISPR Gene Editing Vectors

The pNc-SAG1::CAS9-U6::sgUPRT plasmid (Figure S2) was constructed in our laboratory. It contains the *N. caninum* U6 promoter, Cas9-monomeric enhanced green fluorescent protein (Cas9-mEGFP), 5′UTR and 3′UTR of the *N. caninum* SAG1 gene, single guide RNA sequence (sgRNA), ampicillin resistance gene (AmpR), origin of replication (ori) and f1 ori. To construct the NcGRA6-targeting CRISPR plasmid (pNc-SAG1::CAS9-U6::sgGRA6), we first designed an NcGRA6-specific sgRNA sequence (GGTGACGCTTGTGGCCTTCA) using E-CRISP online software http://www.e-crisp.org/E-CRISP/designcrispr.html. Then, the NcGRA6-specific sgRNA was substituted for UPRT-targeting sgRNA. Specific sgNcGRA6 primers are listed in Table 1. Site-directed mutagenesis was performed using a Q5® Site-Directed Mutagenesis Kit (NEB, Inc., USA) according to the manufacturer's instructions. Step I: The exponential reaction contained 10 ng of pNc-SAG1::CAS9-U6::sgUPRT plasmid, 12.5 μL of Q5 Hot Start High-Fidelity 2 × Master Mix, 1.25 μL of each primer (10 μM), and up to 25 μL of nuclease-free water. After blending and centrifugation, the mixture was immediately subjected to initial denaturation at 98°C for 30 s, followed by 25 amplification cycles of 98°C for 30 s, 55°C for 30 s, and 72°C for 5 min, and a final extension of 72°C for 2 min. Step II: The kinase, ligase, and DpnI (KLD) treatment reaction contained 1 μL of PCR product, 5 μL of 2 × KLD Reaction Buffer, 1 μL of 10 × KLD Enzyme Mix, and 3 μL of nuclease-free water. After mixing, the mixture was incubated at 25°C for 5 min. Step III: The Step II-treated solution was directly used to transform DH5-alpha competent cells. The single colony clones were verified by sequencing (Comate Bioscience Co., Ltd., China) and aligned using DNAMAN.

**Table 1:** Primer sequences used in construction of the *N. caninum* ΔNcGRA6 strain.

**Primer name**	**Sequence (5^**′**^-3^**′**^)**
sgNcGRA6-F^a^	GGTGACGCTTGTGGCCTTCAGTTTTAGAGCTAGAAATAGC
sgNcGRA6-R^b^	AAACAACAATGTCCCTTTGGCA
KO^c^-NcGRA6-F	ATGGCGAACAATAGAACCCTCGCATAAGCTTTACTCGTCGCCAGCAGT
KO^c^-NcGRA6-R	CTATTTTTCCTCCCCGCCGTTTTCGGTCGGAATTTAGGTCGGAAAAGT

a *F represented forward*.

b *R represented reverse*.

c *KO represented knockout*.

#### PCR Amplification of NcGRA6 Donor DNA

The pNcDHFR plasmid was constructed in our laboratory. It contains the 5′UTR and 3′UTR of the *N. caninum* dihydrofolate reductase (DHFR) gene, open reading frame of the mutated DHFR-thymidylate synthase (DHFR-TS), ori and AmpR. To amplify the DHFR-TS DNA donor, specific primers for KO-NcGRA6 were designed (Table 1), and PCR amplification was conducted with Phusion® High-Fidelity DNA Polymerase (NEB, Inc., USA). The PCR reaction contained 200 ng of pNcDHFR plasmid, 0.2 μL of Phusion DNA Polymerase, 4 μL of 5 × Phusion HF Buffer, 0.4 μL of dNTPs (10 μM), 1 μL of each primer (10 μM), and up to 20 μL of nuclease-free water. After mixing, the reaction was immediately subjected to initial denaturation at 98°C for 30 s, followed by 35 amplification cycles of 98°C for 10 s, 55°C for 30 s, and 72°C for 2 min, and a final extension of 72°C for 10 min. The PCR amplification products were purified with a TIANgel Midi Purification Kit (TIANGEN, China).

#### Electroporation

The purified Nc-1 *N. caninum* tachyzoites were filtered through a 5 μm cell filter to remove impurities and debris. The collected supernatants were centrifuged at 1,500 × g for 10 min. Then, the precipitates were resuspended in Cytomix (Thermo Fisher, USA) to a final concentration of 4 × 10^7^ tachyzoites/mL. Next, 2 μg of donor DNA and 6 μg of pNc-SAG1::CAS9-U6::sgGRA6 plasmid were added to 300 μL of the Nc-1 strain, and then, the mixture was transferred into an electroporation cup. To improve electroporation efficiency, the total volume was not more than 350 μL, and the transfection DNA amount ranged between 5 and 10 μg. The electroporation cup was placed into the electric transformation machine and electroporation was conducted at 1,500 V/25 μF/50 Ω. Finally, the collected mixture was inoculated into MDBK cells and cultivated at 37°C in 5% CO_2_ conditions.

#### Screening of the *N. caninum* NcGRA6 Gene Disruption Strain

Tachyzoites were viewed via microscope and collected when 60–80% electroporated *N. caninum* egressed. The egress rate was determined by calculating the average number in 100 randomly selected vacuoles in different areas. The collected tachyzoites were passed through a 27-gauge needle to lyse the cells and release the tachyzoites. Then, HFF cells were inoculated with 2 mL of the harvested tachyzoites and cultivated for 7 h. Pyrimethamine was added into the medium at a concentration of 1 μM and incubated with the cells for 5–7 d for selection pressure during screening. The tachyzoites were harvested when 50% of the tachyzoites egressed. After two screening rounds under pyrimethamine selection pressure, fresh tachyzoites were added into HFF cell cultures in 96-well-plates at 100 μL/well (8–12 tachyzoites/mL) for single tachyzoite screening. The screened single tachyzoites were harvested from the wells when 50% of the tachyzoites egressed and were cultured in 12-well-plates. The screened ΔNcGRA6 tachyzoites were used for further validation.

### Validation of the *N. caninum* ΔNcGRA6 Strain

#### PCR Amplification

**Figure 1:**
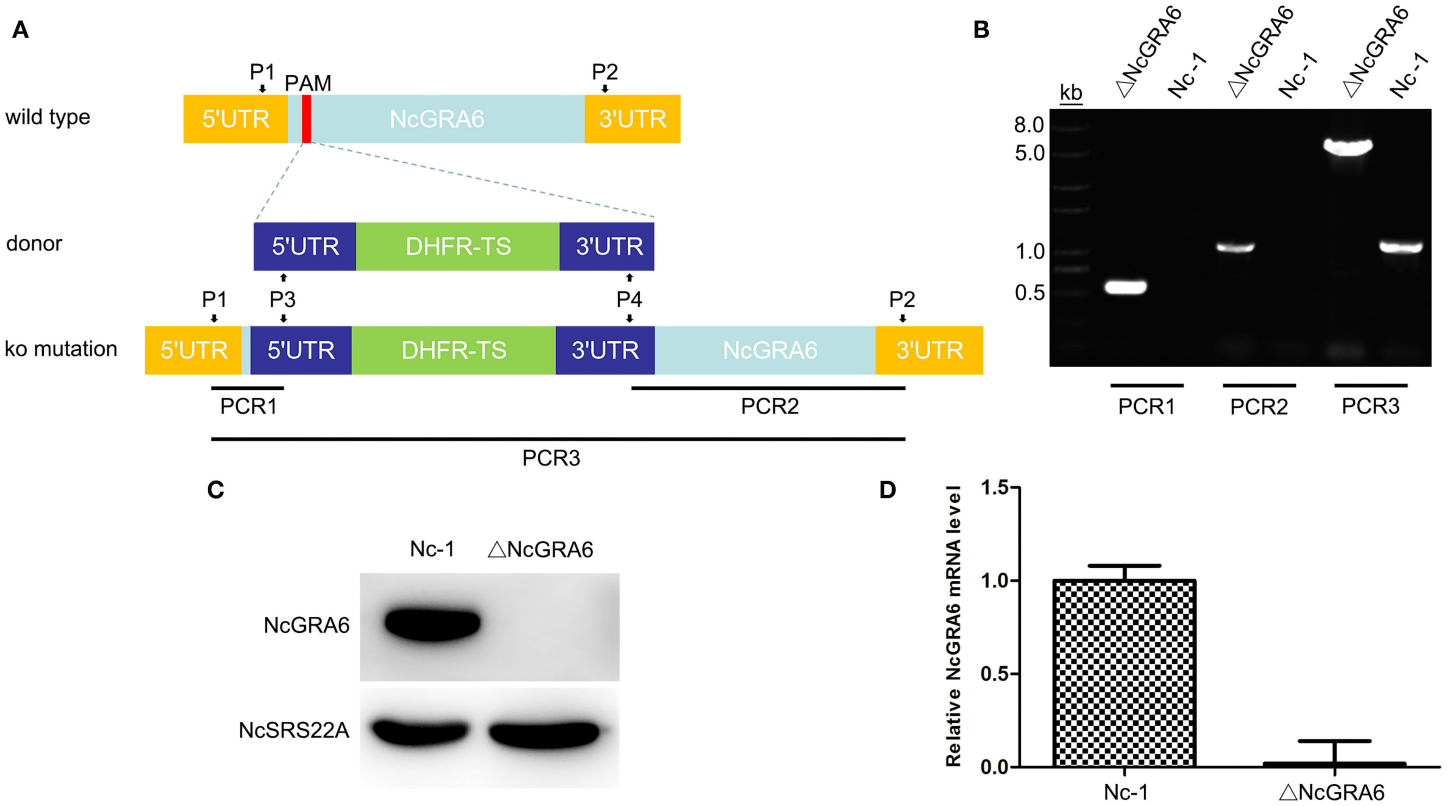
ΔNcGRA6 strain construction schematic diagram and identification of the *N. caninum* ΔNcGRA6 strain using PCR, western blotting, and qPCR. **(A)** *N. caninum* ΔNcGRA6 strain construction schematic diagram and PCR primer design strategies. **(B)** PCR identification of PCR1, PCR2, and PCR3 DNA fragments using parasite genomic DNA. **(C)** Western blot identification of NcGRA6 and NcSRS22A protein levels using corresponding specific antibodies. **(D)** qPCR identification of NcGRA6 gene transcription levels using parasite cDNA.

Genomic DNA was extracted from the ΔNcGRA6 strain and Nc-1 strain (1 × 10^7^ tachyzoites). To screen the ΔNcGRA6 strain at the genomic level, we designed four specific primers (P1–P4) targeting the 5′UTR and 3′UTR of the *N. caninum* SAG1 gene and the 5′UTR and 3′UTR of the *N. caninum* DHFR gene. Three combinations were designed, namely, P1/P3, P2/P4, and P1/P2 (Table 2), to obtain PCR products named PCR1, PCR2, and PCR3 (Figure 1A). PCR1-3 were used to identify whether the DHFR-TS gene had been inserted and the NcGRA6 gene had been disrupted. The PCR reaction system and conditions were the same as those described above. The PCR products were electrophoresed on a 1.5% agarose gel.

**Table 2:** Primer sequences used in validation of the *N. caninum* ΔNcGRA6 strain.

**Primer name**	**Sequence (5^**′**^-3^**′**^)**
P1	AGTTTCCACTGGCTAGTTTCTTGAA
P2	TCCCACAAGTGCATTCTCCAAAC
P3	GAAGCACACGTTTCAGAGACCA
P4	TAGCTCCAGTGTGTCTGTTCCT
NcGRA6-F^a^	ATCCGGTTGAATCCGTGGAG
NcGRA6-R^b^	CTTGTCACGACTGCCTGCTA
Actin-F	TGAGAGAGGATACGGTTT
Actin-R	GGCAGCGGAAGCGCTCGTT

a *F represented forward*.

b *R represented reverse*.

#### Western Blot

To identify the ΔNcGRA6 strain at the protein level, we extracted the protein from the ΔNcGRA6 strain and Nc-1 strain to detect NcGRA6 and NcSRS22A protein levels via western blot analysis. A rat polyclonal antibody against NcGRA6 and a mouse monoclonal antibody against the putative surface antigen NcSRS22A were prepared and constructed in our laboratory. First, the purified ΔNcGRA6 strain and Nc-1 strain tachyzoites were lysed using RIPA buffer (Beyotime, China) supplemented with 1 mM PMSF protease inhibitor. Then, the lysates were mixed with 5 × SDS-PAGE Sample Loading Buffer (Beyotime, China), boiled for 5 min, and electrophoresed on a 12% (w/v) SDS-PAGE gel. After electrophoresis, the protein bands were transferred to polyvinylidene fluoride (PVDF) membranes (Millipore, USA) using a Mini Trans-Blot® 25Electrophoretic Transfer Cell (BIO-RAD, USA). The membranes were blocked with 5% (w/v) skim milk at 4°C overnight, incubated with primary antibody targeting NcGRA6 or NcSRS22A (1:200) for 2 h at 37°C, followed by incubation with HRP-conjugated secondary goat anti-rat IgG (H+L) or goat anti-mouse IgG (H+L) (Earthox, USA, 1:5,000) for 1 h at 37°C, respectively. Finally, the bands were detected using ECL chemiluminescence reagents (Thermo, USA) and visualized with an ECL western blot Detection System (Clinx Science Instruments Co., Ltd., China).

#### RNA Extraction, cDNA Synthesis and Real-Time Quantitative PCR (qPCR)

Total RNA was extracted from 1 × 10^7^ tachyzoites of the ΔNcGRA6 strain and Nc-1 strain using TRIzol reagent (Invitrogen, USA) and dissolved in 10 μL of nuclease-free water. RNA concentration and purity were measured on a Nanodrop ND-2000 apparatus (Thermo Scientific, USA). cDNA was synthesized using 1 μg total RNA with a PrimeScript^TM^ RT Reagent Kit (TaKaRa, China) according to the manufacturer's instructions. To screen the ΔNcGRA6 strain at the transcription level, specific primers for NcGRA6 and actin were designed (Table 2), and qPCR was performed using FastStart Universal SYBR Green Master (Roche, Germany) on a qTOWER 2.0 system (Analytik Jena, Germany). The qPCR reaction contained 1 μL of 20 ng cDNA template, 25 μL of FastStart Universal SYBR Green Master, 1.5 μL of each primer (10 μM), and 21 μL of nuclease-free water. The qPCR reaction conditions were set as follows: denaturation at 95°C for 10 min, followed by 40 cycles of 95°C for 10 s and 60°C for 30 s, and the melting curve was set as the default. Results were normalized to expression of the housekeeping gene actin, and the relative mRNA level was calculated as 2^−ΔΔCt^, where ΔΔCt represents the Ct (sample) - Ct (control).

### Immunofluorescence (IF)

To identify the ΔNcGRA6 strain at the protein distribution level, we located the NcGRA6 protein using an IF assay. MDBK cells were coated on coverslips in 12-well-plates and inoculated with purified ΔNcGRA6 strain or Nc-1 strain tachyzoites. After incubation for 12 h, the cells were fixed in 4% paraformaldehyde solution for 20 min and permeabilized in 0.5% Triton-X-100 for 15 min at room temperature. Subsequently, the cells were blocked with 5% (w/v) BSA at 4°C overnight, incubated with primary antibody targeting NcGRA6 or the putative surface protein NcSRS22A (1:200) in a well for 2 h at 37°C, and then incubated with secondary Cy3-conjugated goat anti-rat IgG (H+L) or FITC-conjugated goat anti-mouse IgG (H+L) (Earthox, USA, 1:400) for 1 h at 37°C. Nuclear DNA was stained with DAPI at a concentration of 300 nM (Thermo, USA). Intracellular parasites were viewed and photographed via laser scanning confocal microscopy (LSCM, Olympus, Japan).

### Immunoelectron Microscopy (IEM)

We also located the NcGRA6 protein using IEM to detect whether NcGRA6 was expressed. The purified ΔNcGRA6 and Nc-1 *N. caninum* strains were inoculated into MDBK cells for 48 h. Then, the cells were scraped, fixed in 4% paraformaldehyde solution for 15 min, dehydrated in ethanol (30–100%) for 10 min, and permeabilized in LR white resin at 20°C overnight. Subsequently, the cells were blocked with 5% (w/v) BSA at 4°C overnight, incubated with primary antibody targeting NcGRA6 (1:40) for 2 h at 37°C, and then incubated with secondary goat anti-rat IgG (H+L) (Thermo Fisher, USA, 1:200) for 1 h at 37°C. Then, the cells were incubated with 10 nm ProteinA-gold and dyed with 2% uranyl acetate. Intracellular parasites were viewed and photographed via transmission electron microscopy (HITACHI, Japan).

### *In vitro* Virulence Assays

#### Plaque Assay

To comprehensively evaluate the virulence of the ΔNcGRA6 strain *in vitro*, a plaque assay was carried out. The purified ΔNcGRA6 strain and Nc-1 *N. caninum* strain were inoculated into HFF cells cultured in 6-well-plates. Each well contained 80–120 tachyzoites. After incubation for 7 d, the cells were fixed in 4% paraformaldehyde for 20 min and stained with 1% crystal violet for 15 min at room temperature. The cells were then washed three times using deionized water and air dried. Finally, plaques were observed and photographed with a fluorescence microscope (Olympus, Japan). The plaque area was measured as previously described (24).

#### Invasion Assay

To explore the invasion ability of the ΔNcGRA6 strain, the purified ΔNcGRA6 strain and Nc-1 *N. caninum* strain were diluted to a final concentration of 1 × 10^6^ tachyzoites/mL and inoculated into HFF cells previously prepared on coverslips in 6-well-plates (100 μL/well) for 20, 40, or 60 min. Then, the cells were fixed in 4% paraformaldehyde for 20 min at RT and permeabilized with 0.1% TritonX-100 for 20 min at RT. Following, cells were blocked with 5% BSA at 4°C overnight and incubated with primary antibodies of NcSRS22A and NcGRA6 for 2 h at 37°C. Finally, cells were incubated with secondary antibodies of FITC-conjugated goat anti-mouse IgG (H+L) and Cy3-conjugated goat anti-rat IgG (H+L) for 1 h at 37°C. Parasites were viewed via a fluorescence microscope. Three independent assays were performed.

#### Egress Assay

To compare the egress ability of the ΔNcGRA6 strain with that of the Nc-1 strain, purified tachyzoites of these two strains were diluted to a final concentration of 1 × 10^6^ tachyzoites/mL and inoculated into HFF cells previously prepared on coverslips in 6-well-plates (100 μL/well) for 1 h. The supernatants were discarded and cells were washed with PBS 3 times. The cells were then cultured in fresh medium for 48 h. Egress was stimulated with the Ca^2+^ ionophore A23187 (Sigma, USA) at 3 μM for 3 min at room temperature. An equal volume of DMSO was added as a negative control. Cells were then fixed in paraformaldehyde, blocked with BSA, and incubated with NcGRA6 primary antibody and Cy3-conjugated goat anti-rat IgG (H+L). Parasites were viewed via a fluorescence microscope. A total of 100 vacuoles were selected in different areas, and the average number was determined. Three independent assays were performed.

#### Replication Assay

The purified ΔNcGRA6 strain and Nc-1 *N. caninum* strain were diluted to 1 × 10^6^ tachyzoites/mL and inoculated into HFF cells previously prepared on coverslips in 6-well-plates (100 μL/well) for 4 h. The supernatants were discarded, and cells were cultured in fresh medium for 6, 12, 24, or 36 h. Then, the cells were fixed in paraformaldehyde, permeabilized in Triton-X-100, blocked with BSA, and incubated with NcSRS22A primary antibody and FITC-conjugated goat anti-mouse IgG (H+L). Parasites were viewed via a fluorescence microscope. A total of 100 vacuoles were selected in different areas, and the average number was determined. Three independent assays were performed.

### *In vivo* Virulence Assays

#### Survival Assay

To compare the virulence of the ΔNcGRA6 strain with that of the parental Nc-1 strain *in vivo*, tachyzoites were intraperitoneally injected into 5-week-old female BALB/C mice (5 mice/group, 2 × 10^7^ tachyzoites/mouse) to construct acute infection models. A PBS-treated group was used as a the negative control. Survival was monitored for 40 d. Two independent assays were performed. According to the 3Rs guidelines, namely, reduction, replacement, and refinement, the mice that were unable to consume food or drink water for over 24 h or lost 20% of body weight were humanely euthanized by cervical dislocation after anesthetization via subcutaneous injection of atropine (0.02 mg/kg).

#### Parasite Burden Determination

Chronic infection models were established. Tachyzoites from the ΔNcGRA6 strain or the parental Nc-1 strain were intraperitoneally injected into 5-week-old female BALB/C mice (9 mice/group, 2 × 10^6^ tachyzoites/mouse). A PBS-treated group was used as a negative control. The mice were euthanized by cervical dislocation after anesthetization via subcutaneous injection of atropine (0.02 mg/kg) at 3 days post infection (dpi), 7, and 15 dpi. Chronically infected heart, liver, spleen, lung, kidney, and brain tissues were removed, and genomic DNA was extracted using a TIANamp Genomic DNA Kit (TIANGEN, China). A standard curve was constructed using a qPCR method based on the *N. caninum*-specific Nc5 gene (Forward: 5′-ACTGGAGGCACGCTGAACAC-3′, Reverse: 5′- AACAATGC TTCGCAAGAGGAA-3′) with genomic DNA of *N. caninum* tachyzoites from 1.0 × 10^2^ to 1.0 × 10^7^ parasites (25). Total DNA (100 ng) from different tissue samples was used as a qPCR template to calculate the parasite burden level in each tissue.

### Histopathological Observation

Heart, liver, brain, and lung tissues were removed and fixed in 4% paraformaldehyde. Then, the tissues were dehydrated with ethanol and dimethyl benzene and embedded in paraffin. The paraffin-embedded tissue was sectioned into 5 μm thick slices and mounted on slides. After paraffin removal and water hydration, the slides were successively treated with hematoxylin solution, acid alcohol, and ammonia solution. Finally, the slides were stained in eosin solution for 5 min, dehydrated, and sealed. Tissue histopathological changes were observed under an optical microscope.

### Statistical Analysis

Statistical analysis was performed using SPAA 18.0 software (SPSS Inc., USA). The results are expressed as Mean ± SD. Comparisons between the Nc-1 strain and ΔNcGRA6 strain were evaluated with a non-parametric test of Mann-Whitney *U-*Test. The graphs were generated in GraphPad Prism 7.00. Significance is indicated by ^*^P < 0.05 and ^**^P < 0.01. No significance is indicated by n.s. Survival was analyzed using a Kaplan Myer survival curve. Histopathology changes were quantified using Image-Pro Plus software.

## Results

### Successful Generation of the *N. caninum* ΔNcGRA6 Strain

Using the *N. caninum* gene editing vector pNc-SAG1::Cas9-U6::sgUPRT as a template, we constructed an NcGRA6-targeting CRISPR plasmid (pNc-SAG1::CAS9-U6::sgGRA6) with NEBuilder® HiFi DNA Assembly Master Mix and a Q5® Site-Directed Mutagenesis Kit. Using the *N. caninum* selectable marker pNc-DHFR plasmid as a template, we designed specific primers to amplify the complete DHFR-TS cassette and added the homologous sequences on both ends of the protospacer-adjacent motif. The constructed pNc-SAG1::CAS9-U6::sgGRA6 plasmid and complete DHFR-TS DNA fragment were co-electroporated into the Nc-1 *N. caninum* strain. Parasites were screened under pyrimethamine selection pressure. Identification of the ΔNcGRA6 line was carried out in the following four aspects: the genome level, transcription level, protein expression level, and distributed location.

The PCR identification is shown in Figure 1B. For PCR1 and PCR2 amplification, the results indicated that the Nc-1 strain had no band, whereas the ΔNcGRA6 strain produced the target 500 and 1,100 bp bands. For PCR3 amplification, the ΔNcGRA6 strain produced a 5,000 bp band, and the Nc-1 strain showed an 1,100 bp band. Through sequencing and alignment, we verified that the ΔNcGRA6 strain was successfully constructed with DHFR-TS gene insertion and NcGRA6 gene disruption.

NcGRA6 gene deficiency was then confirmed using western blotting. The surface antigen protein of NcSRS22A and target protein NcGRA6 were detected. The results showed that the NcSRS22A protein was present in both strains; however, the NcGRA6 protein was only present in the Nc-1 strain (Figure 1C). The results verified that the NcGRA6 protein was absent in the ΔNcGRA6 strain.

The transcription level of the NcGRA6 gene was measured using qPCR. Total RNA was extracted from the ΔNcGRA6 strain, cDNA was synthesized, and the relative mRNA expression level of NcGRA6 was analyzed. The results showed that the NcGRA6 mRNA level was obviously inhibited in the ΔNcGRA6 strain (Figure 1D).

**Figure 2:**
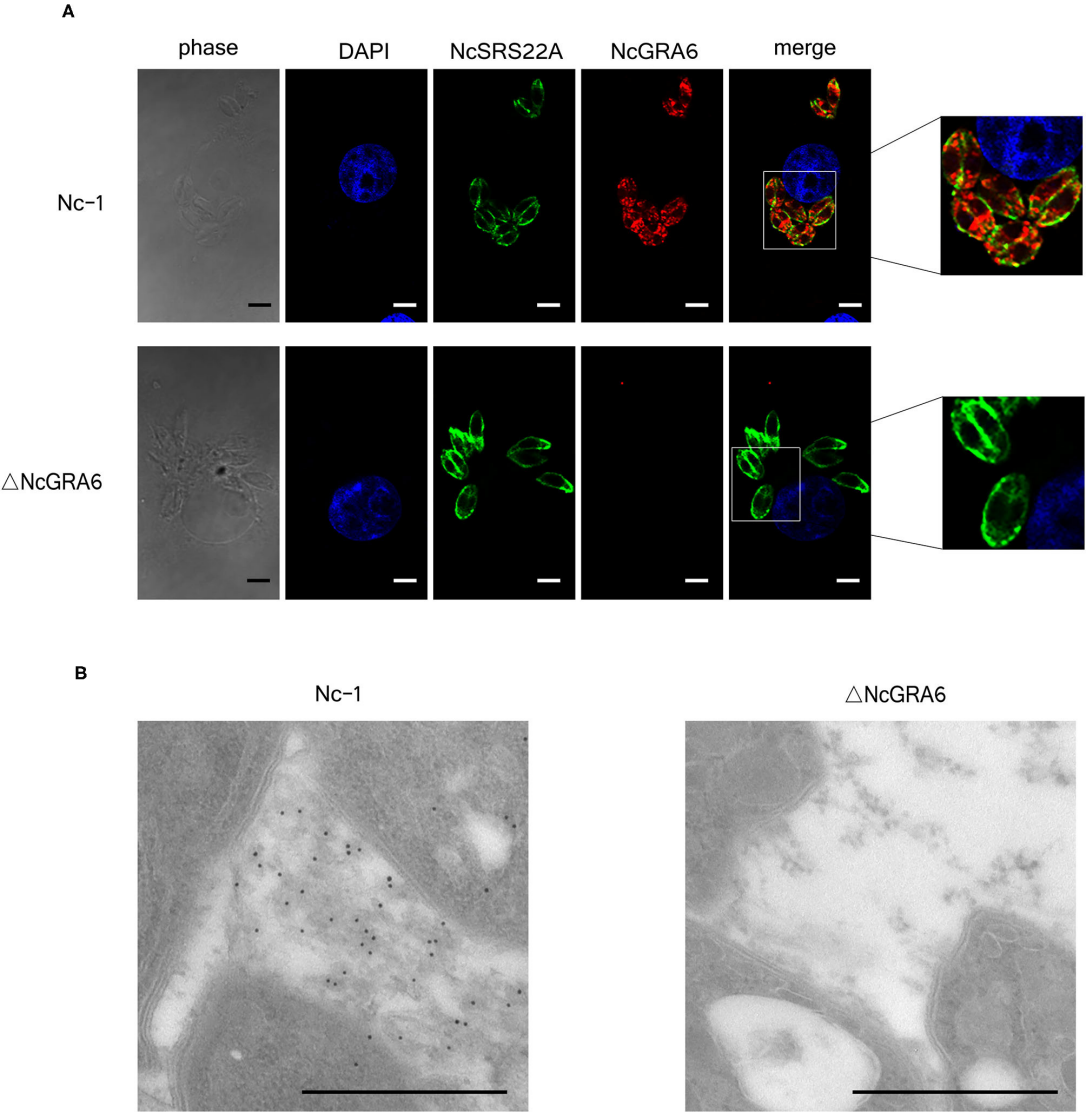
Identification of the *N. caninum* ΔNcGRA6 strain using IF and IEM. **(A)** IF image showing co-localization of NcGRA6 and NcSRS22A proteins in parasite-infected MDBK cells. The NcGRA6 protein was labeled with Cy3, and the NcSRS22A protein was labeled with FITC. Nucleus was stained with DAPI. The merged image integrates DAPI, NcSRS22A and NcGRA6 staining images. The phase image presents the morphology of MDBK cells assessed with a light microscope. **(B)** IEM identification of NcGRA6 protein in parasite-infected MDBK cells. Scale bar, 3.0 μm.

The Nc-1 and ΔNcGRA6 strains were then inoculated into MDBK cells, and subcellular co-localization of NcGRA6 and NcSRS22A proteins was determined using an IF experiment. From Figure 2A, we found that NcSRS22A protein is localized on the surface of both strains; however, only the Nc-1 strain secreted NcGRA6 protein into the PV. These data illustrate that the NcGRA6 protein was not located in the PV of the ΔNcGRA6 strain. An IEM assay revealed that the NcGRA6 protein was located in the IVN structure of the PV after invasion of the Nc-1 strain into the MDBK cells; however, NcGRA6 protein was not observed in the ΔNcGRA6 strain group (Figure 2B). These results suggest that the *N. caninum* ΔNcGRA6 strain was successfully generated.

### The NcGRA6 Gene Is Critical in *N. caninum* Proliferation

**Figure 3:**
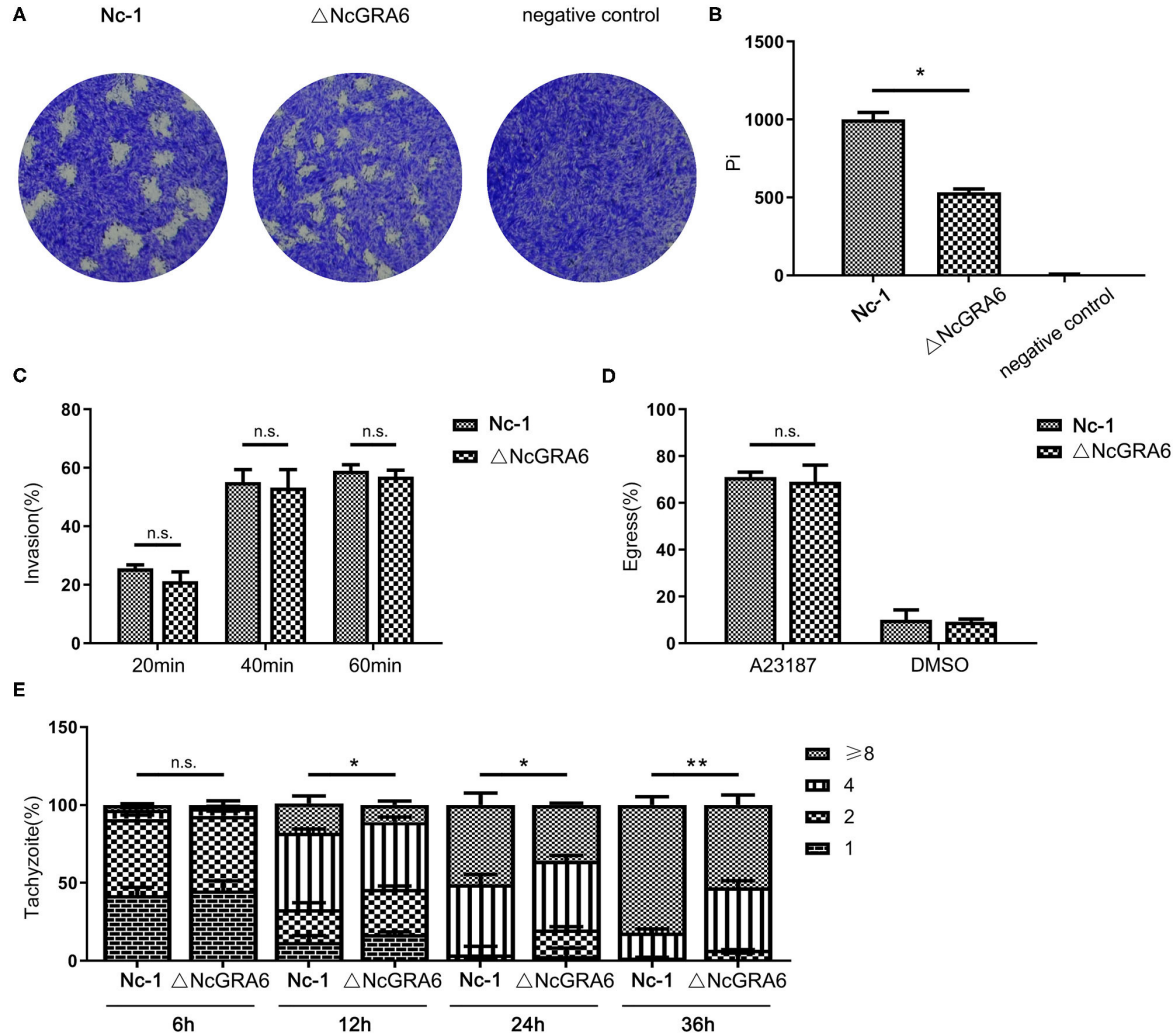
*In vitro* virulence measurement of the *N. caninum* ΔNcGRA6 strain using plaque, invasion, egress, and replication assays. **(A,B)** Virulence was detected using a plaque assay via inoculation of parasites into HFF cells cultured in 6-well-plates (80–120 tachyzoites/well) for 7 d and staining with crystal violet. The negative control was treated with PBS. Pi indicates the number of pixels representing each plaque. **(C)** Virulence was detected using an invasion assay via inoculation of parasites into HFF cells cultured in 6-well-plates (1 × 10^5^ tachyzoites/well) for 20, 40, or 60 min. The IF assay was performed with anti-NcSRS22A and anti-NcGRA6 antibodies. **(D)** Virulence was detected using an egress assay via inoculation of parasites into HFF cells cultured in 6-well-plates (1 × 10^5^ tachyzoites/well) for 48 h and stimulation with the Ca^2+^ ionophore A23187 for 3 min at 3 μM. The solvent for A23187 was DMSO, which was used as the negative control. The IF assay was performed with an anti-NcGRA6 antibody. **(E)** Virulence was detected using an intracellular replication assay via inoculation of parasites into HFF cells cultured in 6-well-plates (1 × 10^5^ tachyzoites/well) for 6, 12, 24, or 36 h. The IF assay was performed with an anti-NcSRS22A antibody. The intracellular vacuoles contained 1, 2, 4, or 8 parasites and are presented by different stripes. *Represents *P* < 0.05, **represents *P* < 0.01, and n.s. represents no significance (Mann-Whitney *U*-Test). Scale bar, 10 μm.

Plaque formation assays can comprehensively reflect of the ability of *N. caninum* to invade host cells, escape from host cells, and reinvade adjacent host cells. During these cycle stages, phenotypic changes at any stage will affect the plaque formation area. Thus, a plaque formation assay was carried out to measure the viability of parasites in HFF cells. Compared with the uninfected negative control group, tachyzoites in both of the inoculated groups displayed obvious, irregular, clear plaque areas against the violet background. In addition, the ΔNcGRA6 strain formed smaller plaques than the Nc-1 strain (Figure 3A). The plaque assay was carried out three times independently. The plaque areas were measured by randomly selecting no <20 plaque fields in every experiment and calculating plaque areas using the pixel points in Photoshop CS5 software (Adobe, USA). As shown in Figure 3B, the pixel number for each plaque (pi) in the ΔNcGRA6 strain was significantly lower than that for plaques in the Nc-1 group (^*^P < 0.05). To explore the exact mechanisms leading to smaller plaques, we further measured the invasion, egress, and proliferation abilities of *N. caninum*. First, we inoculated the ΔNcGRA6 strain and Nc-1 strain into HFF cells for 20, 40, or 60 min and then calculated the number of invasion parasites. There were no significant changes in tachyzoites invasion rate after NcGRA6 gene disruption compared with the Nc-1 strain. Thus, we inferred that the NcGRA6 gene did not influence *N. caninum* invasion ability (Figure 3C). To further study the biological characteristics of ΔNcGRA6 strains, we carried out an egress assay by inoculating tachyzoites into HFF cells and stimulating the cells with a Ca^2+^ ionophore. Meanwhile, a DMSO-treated group was set as a negative control. As shown in Figure 3D, the ΔNcGRA6 strain shared an egress ability similar to that of the Nc-1 strain. This indicates that NcGRA6 gene disruption also had no significant influence on *N. caninum* egress. To investigate whether the NcGRA6 gene is critical in *N. caninum* proliferation, intracellular replication assays were performed via inoculation of HFF cells with tachyzoites for 6–36 h. The number of tachyzoites was counted in each vacuole. Due to the lack of synchronization of host cell invasion, there were 1, 2, 4, 8, or more parasites per intracellular vacuole. The growth ability was about the same in both groups during the initial 6 h. Then, at 12 h, the replication rate in the ΔNcGRA6 strain began to slow compared with that in the Nc-1 group. The number of intracellular vacuoles that contained 1, 2, or 4 parasites in the ΔNcGRA6 group was significantly greater than that in the Nc-1 group (^*^P < 0.05). As the inoculation time lengthened, the difference became increasingly more significant. The distribution of the eight parasites per vacuole was significantly lower for the ΔNcGRA6 strain than for the Nc-1 strain at both 24 h (^*^P < 0.05) and 36 h (^**^P < 0.01). These data indicate that the NcGRA6 gene plays a key role in *N. caninum* replication and show that replication of the ΔNcGRA6 strain was slower than that of the Nc-1 strain (Figure 3E). The results indicate that NcGRA6 expression in *N. caninum* has an important influence on parasite phenotype and is involved in proliferation in host cells.

### Knockout of the NcGRA6 Gene Reduced the Virulence of *N. caninum*

**Figure 4:**
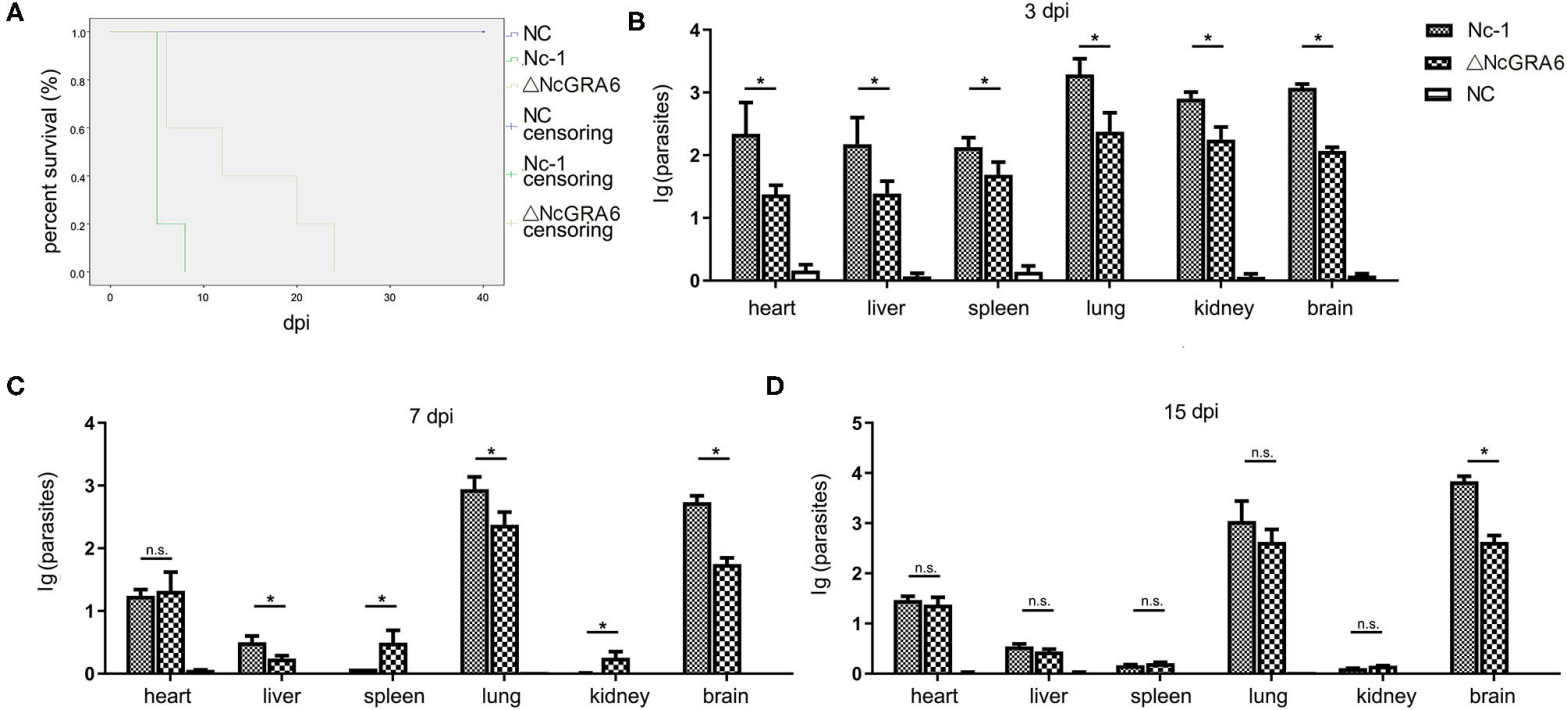
Survival assay measurement and parasite burden determination. **(A)** The *in vivo* virulence of the *N. caninum* ΔNcGRA6 strain was measured using a survival assay in acute infected mice. Five-week-old female BALB/C mice were inoculated with tachyzoites (5 mice/group, 2 × 10^7^ tachyzoites/mouse). **(B**–**D)** The parasite burden in the heart, liver, spleen, lung, kidney, and brain tissues of chronically infected mice at 3, 7, and 15 dpi. Five-week-old female BALB/C mice were inoculated with tachyzoites (9 mice/group, 2 × 10^6^ tachyzoites/mouse). The PBS-treated group was used as a negative control. dpi represented days post infection, *represents *P* < 0.05, and n.s. represents no significance (Mann-Whitney *U-*Test).

Acute infected mouse models were established through inoculation with 2 × 10^7^ tachyzoites/mouse to explore whether NcGRA6 gene disruption effects the survival of infected mice. After inoculation, the mice were observed every day, and we found that all the mice died at day 8 after inoculation with the Nc-1 strain; in contrast, all the ΔNcGRA6-infected mice died at day 24 (Figure 4A). This indicates that the NcGRA6 gene is an important virulence factor and that NcGRA6 gene disruption prolongs the survival of infected mice longer than the Nc-1 strain.

Chronic infected mouse models were established via inoculation with 2 × 10^6^ tachyzoites/mouse. Mouse heart, liver, spleen, lung, and kidney tissues were collected at 3, 7, and 15 dpi. Genomic DNA was extracted, and the parasite burden in each tissue was measured at each timepoint. At 3 days post infection, each tissue had a relatively high parasite burden compared with the PBS-treated negative control group. Moreover, the parasite burden in each tissue from the Nc-1 group was significantly higher than that in tissue from the ΔNcGRA6 group (^*^P < 0.05) (Figure 4B). The parasite burden began to drastically reduce as the infection period increased, except in lung and brain tissues. In heart tissues, the parasite burden was approximately the same at both 7 and 15 dpi. In liver tissues, the parasite burden was significantly lower in the ΔNcGRA6 group than in the Nc-1 group at 7 dpi (^*^P < 0.05). However, at 15 dpi, there was no significant difference in liver tissues between these two groups. In spleen and kidney tissues, only a few parasites could be detected after 15 dpi, and no significant difference was observed. It is worth mentioning that the parasite burden in the lung and brain tissues was always at high levels during the monitored 15 days. However, the parasite burden in the ΔNcGRA6 group was still significantly lower than that in the Nc-1 group, especially in brain tissues (^*^P < 0.05) (Figures 4C,D). The results suggest that the parasite burden was high in brain, lung, heart, and liver tissues, especially in the brain and lung tissues. Moreover, NcGRA6 gene disruption lowered the parasite burden in target tissues compared with the Nc-1strain.

**Figure 5:**
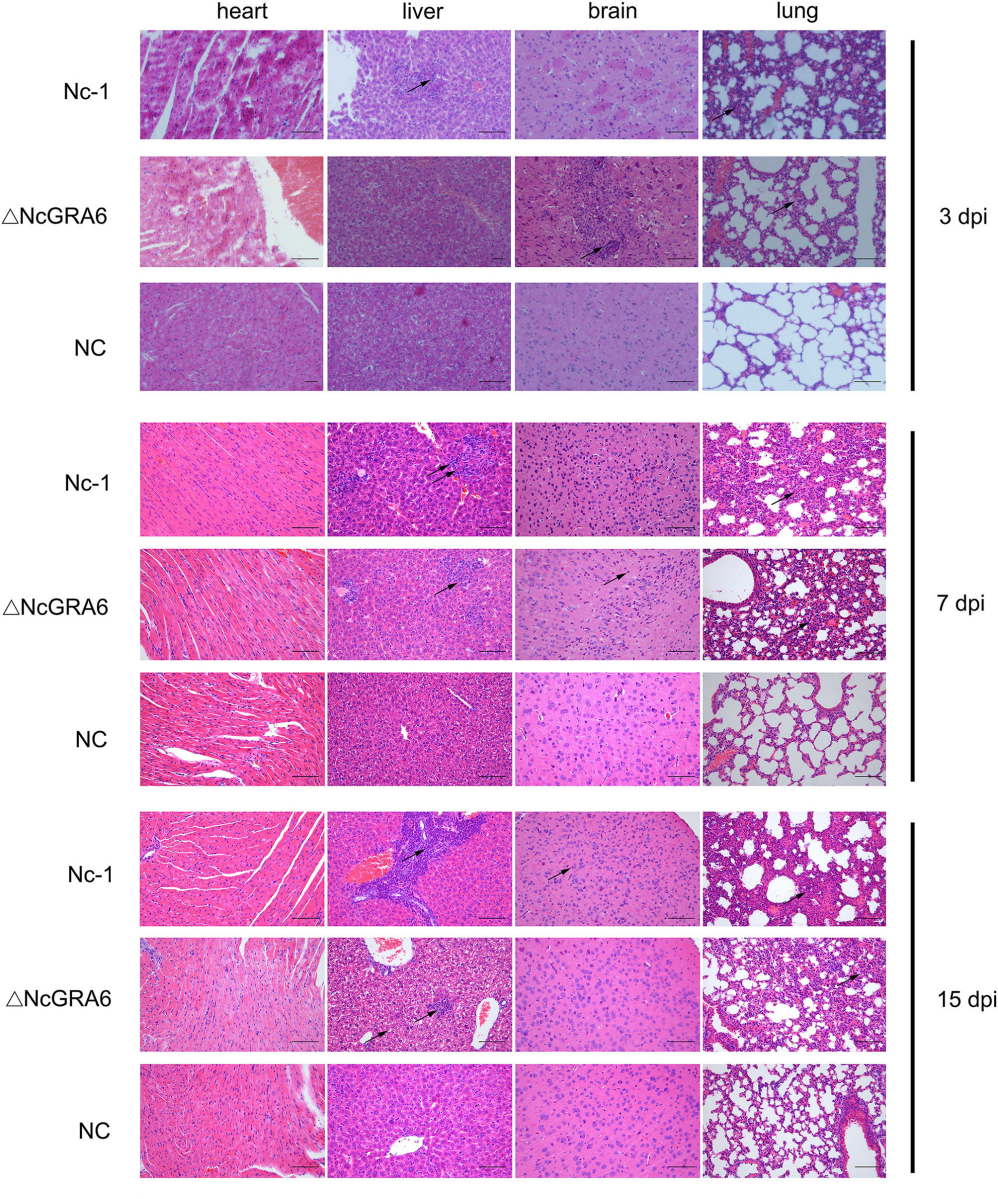
Histopathological changes in chronic infection models. Five-week-old female BALB/C mice were inoculated with tachyzoites (9 mice/group, 2 × 10^6^ tachyzoites/mouse). Mouse heart, liver, brain, and lung tissues were separated, and HE staining was carried out to observe the histopathological changes at 3, 7, and 15 dpi. The PBS-treated group was used as a negative control. Bar = 100 μm. Arrows indicate typical histopathological changes.

**Figure 6:**
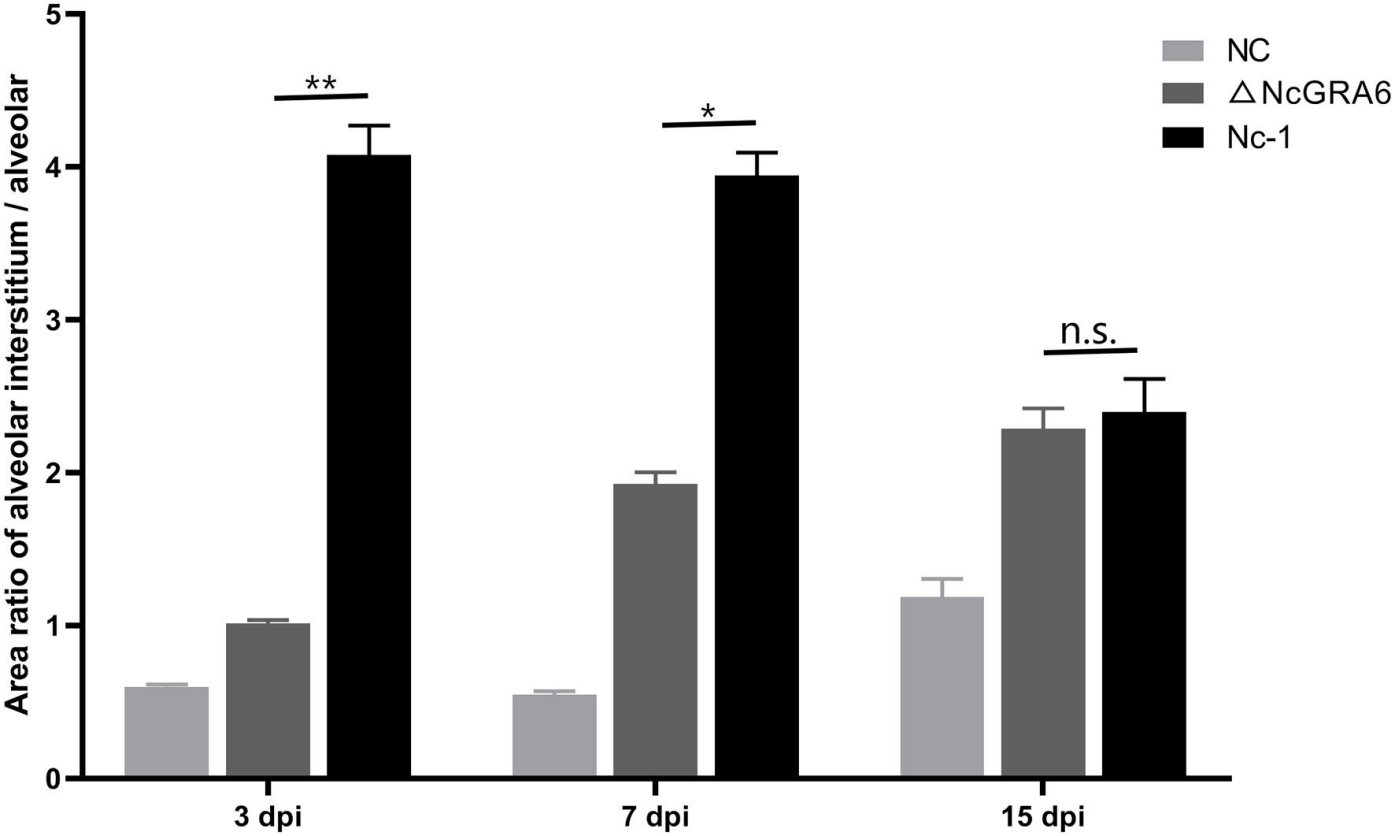
Quantitation analysis of the histopathological changes in lung tissues. Pathology changes of HE stained lung tissues were quantified by calculating the area ratio of alveolar interstitium / alveolar to compare the pathology changes among NC, ΔNcGRA6, and Nc-1 groups. *P < 0.05, **P < 0.01, and n.s. (Mann-Whitney *U-*Test).

Subsequently, we observed histopathological changes in parasite-intensive tissues of the brain, lung, heart, and liver at 3, 7, and 15 dpi. At 3 dpi, obvious changes were observed in lung tissues. The ΔNcGRA6 group exhibited a less thickened alveolar mass and less inflammatory cell infiltration compared with the Nc-1 group. Moreover, there was significant inflammatory cell infiltration in the Nc-1 group compared with the ΔNcGRA6 group and negative control group. At 7 dpi, mice inoculated with the Nc-1 group showed moderately widened alveolar interstitium and hyperplasia of alveolar epithelial cells; small necrotic foci in hepatic lobules, with inflammatory cell infiltration; and glial hyperplasia with a small amount of macrophage infiltration. For the ΔNcGRA6 group, histopathological changes included slightly widened alveolar interstitium, inflammatory cell foci infiltration in the liver, and slight local glial cell hyperplasia in the brain. At 15 dpi, mice inoculated with the Nc-1 group exhibited significantly widened alveolar interstitium with a small amount of neutrophils infiltration; a large number of inflammatory cells in the hepatic portal area; and mildly proliferating glial cells. For the ΔNcGRA6 group, the histopathological changes included slightly widened alveolar interstitium and hepatocyte degeneration, with partial small necrotic foci and inflammatory cell infiltration (Figure 5). Overall, as a target organ, the lung displayed obvious histopathological changes throughout the acute infection period. Both the ΔNcGRA6 group and NC group showed higher alveolar interstitial/alveolar area ratio than the negative control group. Moreover, both the ΔNcGRA6 group and Nc-1 group showed a increased alveolar interstitial/alveolar area ratio with an increase in infection time. The differences between the ΔNcGRA6 group and Nc-1 group were significant at 3 dpi (^**^P < 0.01) and 7 dpi (^**^P < 0.05) but not significant at 15 dpi (Figure 6). These data illustrate that the NcGRA6 gene may play important roles in the pathogenicity of *N. caninum* toward hosts cells, especially in the initial infection stage.

## Discussion

After invasion of host cells, *N. caninum* multiplies within the PV and absorbs nutrients and eliminate wastes through the IVN (9). *Toxoplasma gondii* (*T. gondii*) is the model organism used to study *Apicomplexa* protein functions (26). It is reported that GRA proteins are the core components in forming the PV and IVN (15). In our previous *N. caninum* study, we found that the NcGRA6 protein exists in both soluble and membrane-associated forms. The soluble form is located in the PV lumen. The membrane-associated form is located in the IVN of the PV (20). For *T. gondii*, the IVN is replaced by small vesicles within the vacuolar space in the case of TgGRA6 gene deletion (16). Thus, we can infer that the GRA6 gene is one of the most important virulence factors for *N. caninum* and that GRA6 gene disruption may influence formation of the PV and IVN. Whether GRA6 gene disruption affects the proliferation of tachyzoites and the survival of host cells is still unknown for *N. caninum*. Thus, the present study aimed to explore new targets for developing vaccines against neosporosis by establishing an *N. caninum* ΔNcGRA6 strain based on the Nc-1 strain and studying its virulence *in vitro* and *in vivo*.

CRISPR is a DNA recognition system used as a defense mechanism in bacteria and archaea. Modification of the CRISPR system has made gene disruption available in a variety of organisms with high efficiency and has facilitated exploration of the roles of gene functions (27–29). CRISPR/CAS9 technology was first applied to the model organism *T. gondii* in 2014 and found to include many ways to repair double-strand breaks, including methods used in homologous recombination and non-homologous recombination. At present, only two studies have used CRISPR/CAS9 technology to disrupt genes in *N. caninum*. Arranz-Solís et al. directly used the *T. gondii* CRISPR/CAS9 gene editing system to disrupt the NcGRA7 gene in the Nc-Spain7 isolate (30). At first, we attempted to use the *T. gondii*-specific genome-editing pSAG1::CAS9-U6::sgUPRT system to disrupt NcGRA6; however, we found that the lower gene knockout efficiency in *N. caninum* made it difficult to obtain a stable ΔNcGRA6 strain. Yang et al. (31) replaced the U6 promoter in *T. gondii* CRISPR/CAS9 with the *N. caninum* U6 promoter and successfully generated an NcGRA17 knockout strain. We think that not only the *N. caninum*-specific U6 promoter, but also SAG1 promoter and terminator, and DHFR promoter and terminator are all important to improve the gene knockout efficiency in *N. caninum*. Subsequently, we sought to construct an *N. caninum*-specific pNc-SAG1::CAS9-U6::sgUPRT genome-editing plasmid (data not published, Figure S2) based on pSAG1::CAS9-U6::sgUPRT (32) and constructed an NcGRA6-targeting CRISPR plasmid (pNc-SAG1::CAS9-U6::sgGRA6). The CRISPR/CAS9 system disrupted the NcGRA6 gene via insertion of an *N. caninum*-specific DHFR-TS marker. Then, the pNc-SAG1::CAS9-U6::sgGRA6 plasmid and selectable marker of DHFR-TS were electroporated into the Nc-1 strain, which was inoculated into HFF cells and allowed screening under pyrimethamine selection pressure. It is worth mentioning that gene knockout using stable transfection can avoid the common off-target effects brought by transient transfection using the RNAi method (33). In addition, the gene knockout method can completely remove the function of GRA6; however, the RNAi method can only lower the function of GRA6 to some extent, which may influence the following function research of GRA6. Although the gene knockout method involves high difficulty, a complicated procedure, and a long experimental time, it still displayed excellent advantages for gene function research. After identification at the genomic level, protein level, transcription level, and protein distribution, we verified that the ΔNcGRA6 strain was successfully generated and could be used for further NcGRA6 gene function research. In *N. caninum*, the NcSRS2 protein was used as a surface protein in GRA17 and ROP5 gene knockout strains (31, 34). During identification of the ΔNcGRA6 strain at the protein level, we first labeled the specific putative surface protein NcSRS22A in *N. caninum* using its monoclonal antibody (identified and prepared in our laboratory) to explore the location of GRA6 protein. Compared with the PV labeling method, we were not only able to determine the exact location and distribution of GRA6 in *N. caninum* but also the location between tachyzoites in the PV. However, it is difficult to confirm whether NcGRA6 is secreted into the lumen of the PV if the PV is labeled rather than the tachyzoites. Overall, the present study constructed an *N. caninum* CRISPR/CAS9 gene editing system to efficiently edit *N. caninum* and provides a reference method and lays a foundation for further studies on other *N. caninum* proteins.

Previous studies have identified several GRA proteins, such as GRA6 and GRA2, that play roles in *T. gondii* virulence during acute infection using type I parasites (16, 35). In detail, the N-terminal domain of GRA6 was found to be associated with the IVN, which is involved in *T. gondii* virulence. Disruption of TgGRA6 disrupts the structure of the IVN (16, 36). To further explore the influence of NcGRA6 in *N. caninum* virulence, we conducted experiments both *in vitro* and *in vivo*. Plaque, invasion, egress, and replication assays are common technologies for the applied in *in vitro* parasite virulence research (31, 34). In our study, we performed these assays to compare virulence changes between the *N. caninum* ΔNcGRA6 strain and Nc-1 strain. The reverse genetic studies confirmed that NcGRA6 disruption did not affect the invasion and egress ability of the *N. caninum* tachyzoites but did affect the proliferation in host cells. We wondered whether NcGRA6 disruption could lead to similar results *in vivo*. Thus, we established a chronic infection mouse model using a low dose tachyzoites (2 × 10^6^ per mouse) with the aim of detecting the parasite burden. In *T. gondii*, a TgGRA6 gene knockout strain was constructed using type II *T. gondii*, and the cysts in the brain were detected. The brain cyst burden in TgGRA6 gene knockout strain-infected mice was significantly reduced in comparison with that in wild-type parasite-infected mice (37). Based on the parasite burden at 3 dpi, we also found that NcGRA6 depletion severely influenced *N. caninum* proliferation; however, the influence was reduced with increased infection time. The parasite burden difference was significantly reduced between the Nc-1 group and ΔNcGRA6 group. These data indicate that NcGRA6 depletion was the major cause of the slowdown of *N. caninum* proliferation at the early infection period. Further, we considered whether NcGRA6 depletion could change the pathogenicity to hosts. Thus, we increased the infection dose 10-fold to 2 × 10^7^ tachyzoites per mouse to establish an acute infection model. At the early infection period, the survival rate in the ΔNcGRA6 group was obviously higher than that in the Nc-1 group. However, all the mice died over time. These data also demonstrate that the virulence of NcGRA6 mainly functioned during the early infection stage. To explore the major reason why NcGRA6 depletion delayed death in mice, we carried out a histopathological examination, confirmed that the lung was the target organ, and found that NcGRA6 depletion could reduce the damage to the target organ. In summary, NcGRA6 disruption slowed *N. caninum* proliferation and lowered the pathogenicity to hosts both *in vivo* and *in vitro*.

## Conclusions

We successfully generated an NcGRA6 gene disruption strain using a CRISPR/Cas9-directed genome editing system specifically for *N. caninum*. Biological function studies on the ΔNcGRA6 strain indicated that GRA6 gene disruption slowed *N. caninum* proliferation and reduced its virulence to hosts. Our findings here highlight the role of GRA6 as a virulence factor in *N. caninum* and lay a foundation for exploring the interaction mechanisms between parasites and hosts with the aim of controlling neosporosis.

## Data Availability Statement

All datasets presented in this study are included in the article/Supplementary Material.

## Ethics Statement

The animal study was reviewed and approved by Animal Welfare and Research Ethics Committee of Jilin University (pzpx20190929065).

## Author Contributions

PZ designed the research. PZ, NZ, and JD conducted the research. JL, XW, XinL, XiaL, and JY analyzed the data. PZ, JD, and XZ wrote the manuscript. XZ and PG directed the project. All authors have read and approved the manuscript.

## Conflict of Interest

The authors declare that the research was conducted in the absence of any commercial or financial relationships that could be construed as a potential conflict of interest.

## Supplementary Material

Figure S1 Homology analysis of GRA6 gene sequences in Nc-1 strain and Nc-liv strain.

Figure S2 Plasmid profile of pNc-SAG1::CAS9-U6::sgUPRT.
