# Supplementary figures and images for: Effects of Dense Granular Protein 6 (GRA6) Disruption on Neospora caninum Virulence
Source: Front Vet Sci. 2020 Sep 22;7:562730. doi: 10.3389/fvets.2020.562730 (PMC7536263; doi:10.3389/fvets.2020.562730)

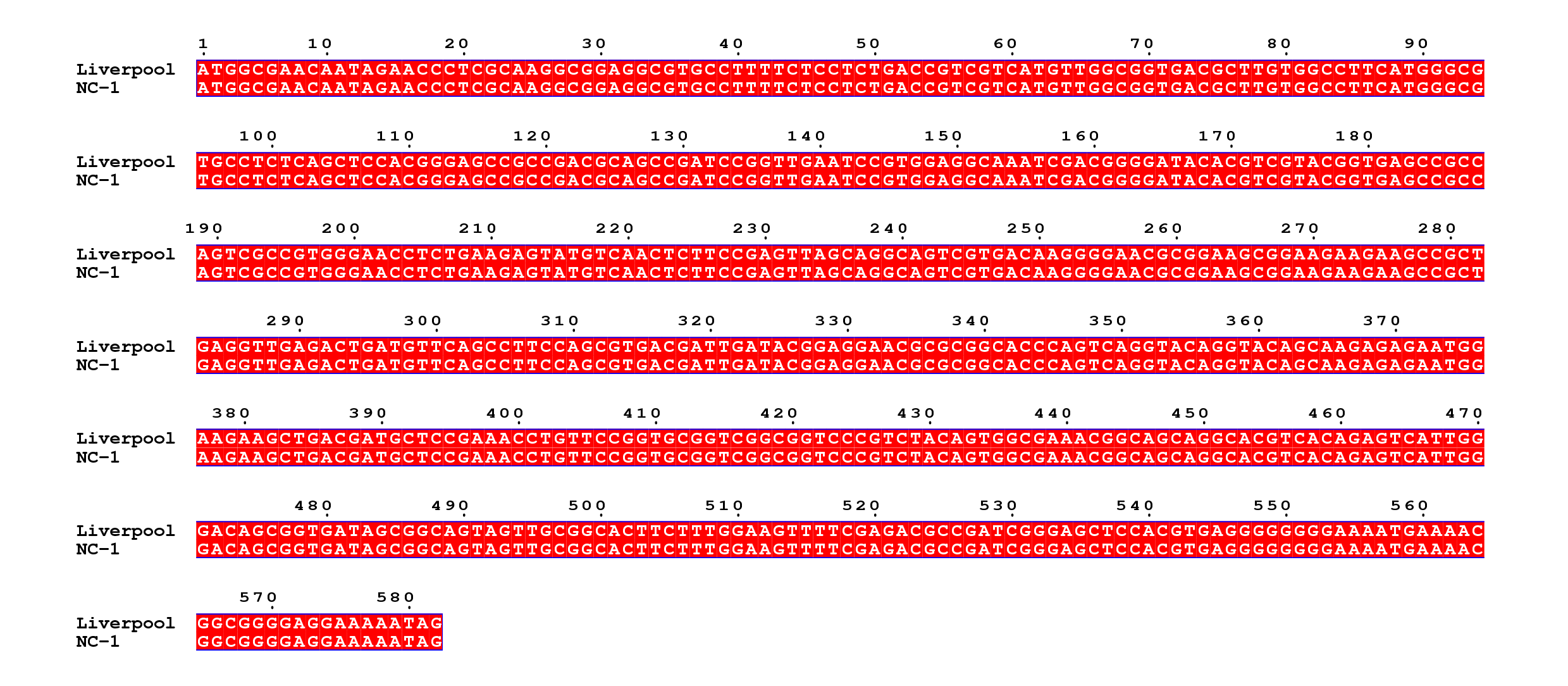

Supplement: Figure S1 — Homology analysis of GRA6 gene sequences in Nc-1 strain and Nc-liv strain. [file Image_1.TIF]

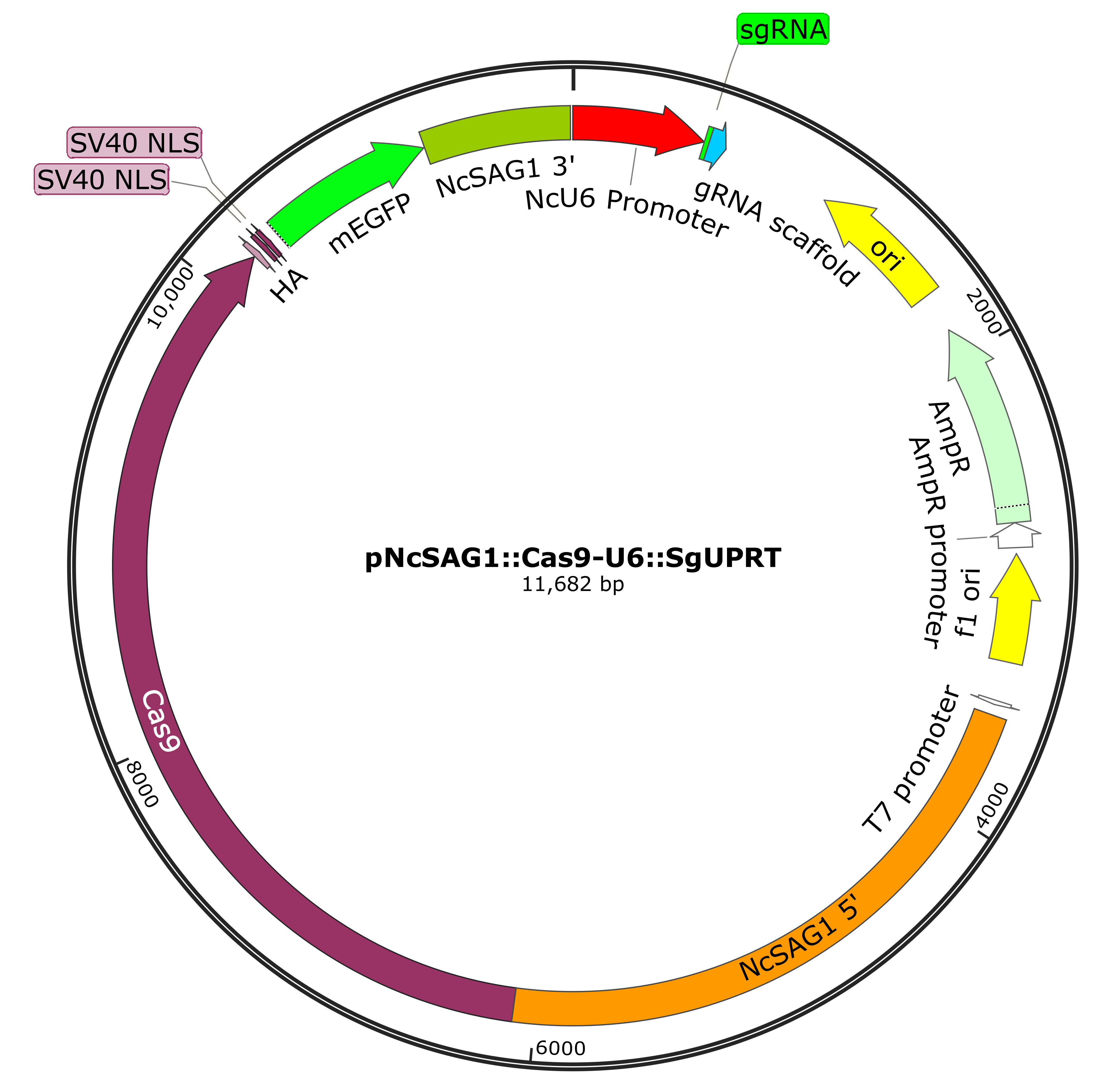

Supplement: Figure S2 — Plasmid profile of pNc-SAG1::CAS9-U6::sgUPRT. [file Image_2.TIFF]
